# Pathological similarities between degenerative enthesopathy and rheumatic enthesitis: the role of ECM stiffness and Wolff's Law-based mechanical stimulation as a therapeutic strategy

**DOI:** 10.3389/fresc.2026.1716050

**Published:** 2026-08-20

**Authors:** Kang Ahn, Aerisa Ahn, Seung Jun Oh, Hyun Sung Kim

**Affiliations:** 1 Department of Pain and Rehabilitation, Ahnkang Hospital, Seoul, Republic of Korea; 2 Graduate School of Medicine, CHA University, Seongnam-si, Gyeonggi-do, Republic of Korea; 3 Department of Physical Therapy, Sangji University, Wonju-si, Gangwon-do, Republic of Korea

**Keywords:** dry needling, enthesis, enthesopathy, extracellular matrix stiffness, mechanotransduction, vibration therapy

## Abstract

Degenerative enthesopathy and rheumatic enthesitis have traditionally been regarded as distinct disorders driven by mechanical overload and immune dysregulation, respectively. However, increasing evidence suggests that the mechanobiological links between these conditions have been underappreciated. In particular, pathological stiffening of the extracellular matrix (ECM) at the enthesis has emerged as a potential common mechanism connecting cumulative mechanical loading, chronic low-grade inflammation, and dysregulated mechanotransduction. This review synthesizes current biomechanical and immunological evidence linking ECM rigidity to fibrocartilage metaplasia, bone marrow edema, and pathological neo-osteogenesis. Histopathological similarities between degenerative and inflammatory phenotypes, including subperiosteal fibrosis, advancing mineralization fronts, and immune-cell trafficking through transcortical vessels (TCVs), further support the concept of a shared disease continuum. ECM stiffness may represent a shared mechanobiological feature contributing to structural degeneration, chronic inflammation, and aberrant ossification in both conditions. We further examine the therapeutic potential of Wolff's Law-based mechanical stimulation strategies, including dry needling, vibration therapy, and targeted mechanical stimulation of periosteal and entheseal regions. These interventions engage mechanosensitive pathways that may restore matrix compliance, improve microvascular perfusion, and modulate inflammatory activity within the enthesis microenvironment. Taken together, the available evidence indicates that ECM stiffness is not only a marker of disease progression but also a plausible therapeutic target. This perspective may inform future stiffness-oriented treatment strategies, including bio-electric stimulation-based approaches, for restoring enthesis homeostasis in both degenerative and rheumatic disorders.

## 1 Introduction

Tissue mechanics play a pivotal role in regulating cellular behavior and maintaining physiological homeostasis. In recent years, the extracellular matrix (ECM) has emerged as a central determinant of tissue function, acting not only as a passive scaffold but also as an active regulator of cell fate through its mechanical properties (1–3). Among these properties, ECM stiffness has been increasingly recognized as a key pathogenic factor in a range of degenerative and inflammatory diseases, including osteoarthritis (OA), tendinopathy, fibrosis, and sarcopenia (4, 5). Beyond its structural role, ECM stiffness is increasingly recognized as a dynamic mechanobiological regulator capable of modulating immune activation, stem cell differentiation, and tissue remodeling across musculoskeletal diseases.

At the intersection of biomechanics and pathology lies the enthesis—the specialized tissue where tendons, ligaments, fascia, and joint capsules attach to bone. This complex transitional structure plays a critical role in load transmission and mechanical buffering, but is also uniquely susceptible to structural deterioration under repetitive or dysregulated mechanical stress. Pathological changes at this interface, collectively termed enthesopathy, can arise from both immune-mediated (enthesitis) and mechanical (degenerative) etiologies. Despite differing upstream triggers, both forms share common downstream mechanisms, including aberrant matrix remodeling, fibrocartilage proliferation, and subchondral bone sclerosis. However, despite these pathological overlaps, degenerative enthesopathy and rheumatic enthesitis have traditionally been investigated as distinct clinical entities, resulting in limited integration between mechanobiological and immunological perspectives.

Degeneration and inflammation likely contribute to unresolved enthesopathy and related joint diseases, such as osteoarthritis, tendinopathy, and rotator cuff disease (6–8). Hallmark characteristics of unresolved tendinopathy and enthesopathy include neovascularization and increased innervation of the tendon and enthesis (8). Rheumatological conditions, such as fibromyalgia and psoriasis, can also manifest entheseal changes and damage which are likely not purely mechanically derived. Importantly, both degenerative enthesopathy and rheumatic enthesitis are recognized to involve chronic inflammation as a central driving factor (9). This inflammation may be initiated by mechanical microdamage or immune dysregulation, leading to a sustained inflammatory environment that disrupts the structural and functional integrity of the enthesis. One of the key structures affected in this process is the Sharpey's fibers, which are collagenous fibers that anchor the periosteum and tendons into cortical bone. Inflammatory degeneration of Sharpey's fibers weakens the mechanical continuity between soft tissue and bone, impairing load transfer and facilitating progressive ECM stiffening and ossification at the enthesis (10, 11).

ECM stiffening at the enthesis—driven by collagen crosslinking enzymes such as lysyl oxidase (LOX), dysregulated mechanotransduction, and chronic inflammation—contributes to pain, reduced tissue compliance, and impaired repair capacity (12, 13). Notably, the biomechanical environment of the enthesis is governed by Wolff's Law, wherein mechanical loading directly influences the structure and density of adjacent bone and soft tissue. This principle suggests that mechanical input is not merely a contributor to pathology but a potential therapeutic vector. Although recent studies have independently explored inflammatory signaling, ECM remodeling, and mechanotransduction in enthesis disorders, few reviews have comprehensively examined how ECM stiffness may serve as a shared pathological substrate linking degenerative and inflammatory enthesopathies.

Recent studies have recontextualized mechanical stress as a modifiable and regenerative force capable of reversing ECM fibrosis and restoring compliance. Modalities such as low-intensity pulsed ultrasound (LIPUS), therapeutic stretching, and periosteal dry needling have demonstrated the ability to modulate mechanosensitive signaling pathways (e.g., YAP/TAZ, Piezo channels), promote collagen turnover, and recondition fibrotic tissue (14–16).

This review aims to critically examine the role of mechanical stress in enthesis pathology, with a particular focus on ECM stiffness as a convergent mechanism underlying both degenerative enthesopathy and rheumatic enthesitis. By integrating insights from mechanobiology, regenerative rehabilitation, and matrix-targeted therapeutics, we propose a framework in which mechanical modulation is leveraged not only for symptom relief but also for structural restoration of the tendon–bone interface.

Unlike previous reviews that primarily focused on inflammatory pathways or degenerative structural changes independently, this review emphasizes the shared mechanobiological convergence between these conditions and highlights ECM stiffness as both a pathological driver and a therapeutic target.

Furthermore, we discuss how Wolff's Law-based mechanical stimulation strategies may provide a regenerative approach capable of interrupting the stiffness–inflammation cycle characteristic of chronic enthesis disorders.

## 2 Mechanical stress and ECM stiffness: initiation of pathophysiology

### 2.1 Interplay between mechanical damage and ECM rigidity

**Figure 1:**
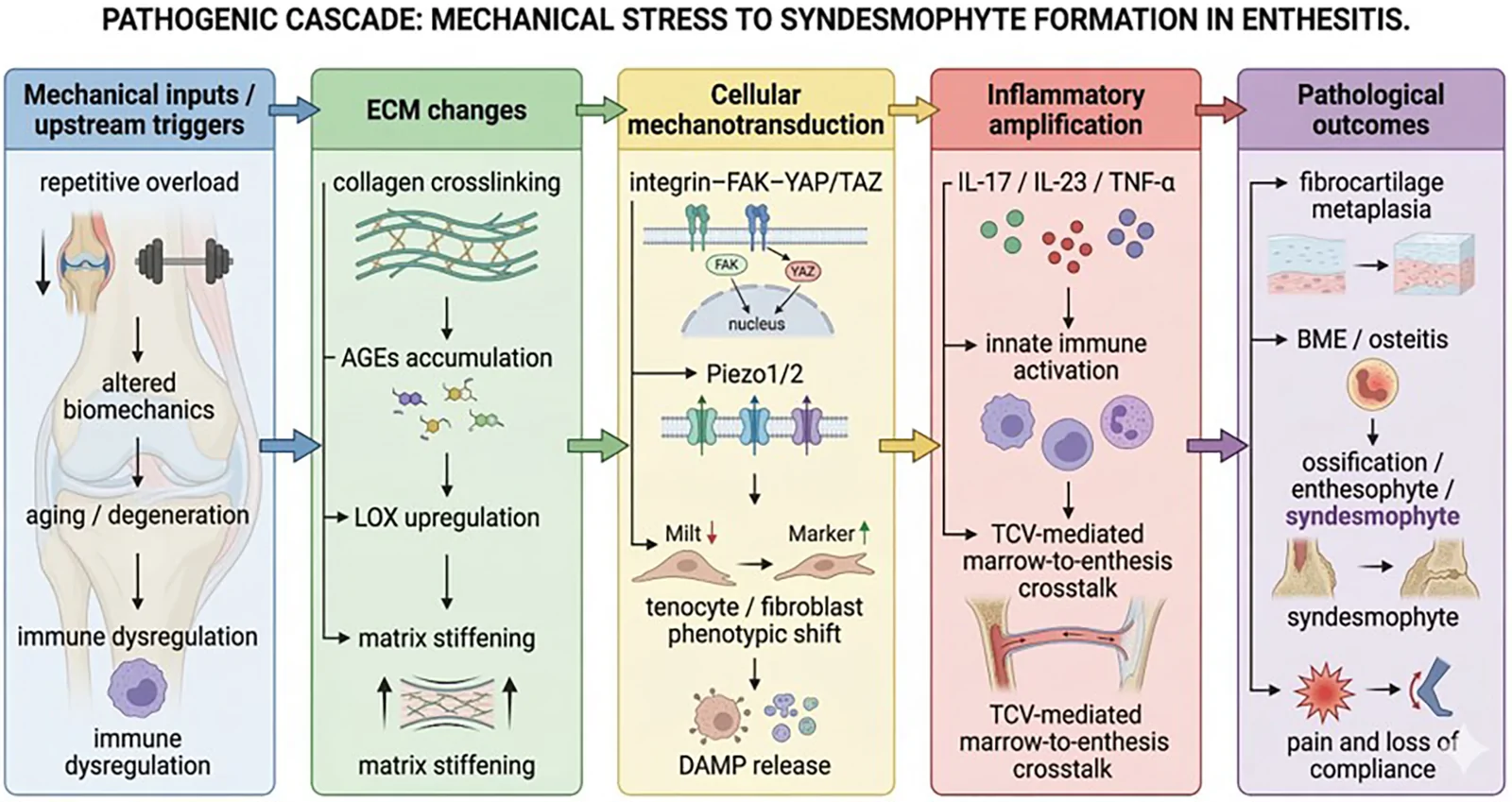
Unified mechanobiological model linking mechanical stress, ECM stiffening, cellular responses, and pathological remodeling at the enthesis.

At the tendon–bone interface, or enthesis, the ECM functions not only as a mechanical anchor but also as a critical regulator of local cellular behavior through its biochemical and biomechanical properties. Repetitive or excessive mechanical loading from overuse, altered biomechanics, or age-related degeneration leads to progressive structural disruption of the enthesis and the surrounding ECM (Figure 1). This includes reduced elasticity, increased collagen cross-linking, and accumulation of Advanced Glycation End-products (AGEs), resulting in a progressively stiffer matrix (17).

Stiffened ECM disrupts mechanotransduction pathways such as integrin–FAK–YAP/TAZ and Piezo1/2-mediated calcium signaling, skewing fibroblast and tenocyte behavior toward pro-fibrotic and inflammatory phenotypes (18). These changes accelerate the deposition of type III collagen, increase LOX activity, and impair matrix turnover through dysregulation of MMPs and TIMPs (13, 19) (Figure 1). This pathological remodeling reduces the biomechanical compliance of the enthesis and increases the risk of microtearing, peri-entheseal ossification, and degenerative tissue failure—hallmarks of degenerative enthesopathy.

From the perspective of Wolff's Law, mechanical stress typically induces adaptive remodeling in healthy tissues. However, when mechanical signals exceed physiological thresholds or persist under maladaptive loading patterns, they contribute to enthesis degradation rather than regeneration. This transformation from adaptive to pathological remodeling underscores the dual role of mechanical forces in musculoskeletal health and disease. Importantly, this shift from adaptive loading to pathological mechanotransduction may represent a critical early event linking mechanical microdamage to chronic inflammatory remodeling at the enthesis.

### 2.2 Immune response similarities

Although degenerative enthesopathy and rheumatic enthesitis arise from distinct etiologies, the two conditions converge at both structural and immunological levels. Mechanical microdamage in the enthesis has been shown to act as a potent stimulus for innate immune activation. Benjamin et al. (20) proposed that enthesis-resident cells respond to mechanical stress via damage-associated molecular patterns (DAMPs), initiating immune responses even in the absence of infection or systemic autoimmunity (20).

Recent evidence reveals that degenerative enthesopathy exhibits low-grade, non-resolving inflammation with elevated expression of IL-17, IL-23, and TNF-α—cytokines traditionally associated with rheumatic diseases such as ankylosing spondylitis and psoriatic arthritis (21). This suggests that mechanical overload and ECM stiffening can recapitulate key features of immune-mediated enthesitis, establishing a common inflammatory microenvironment despite differing upstream triggers.

**Figure 2:**
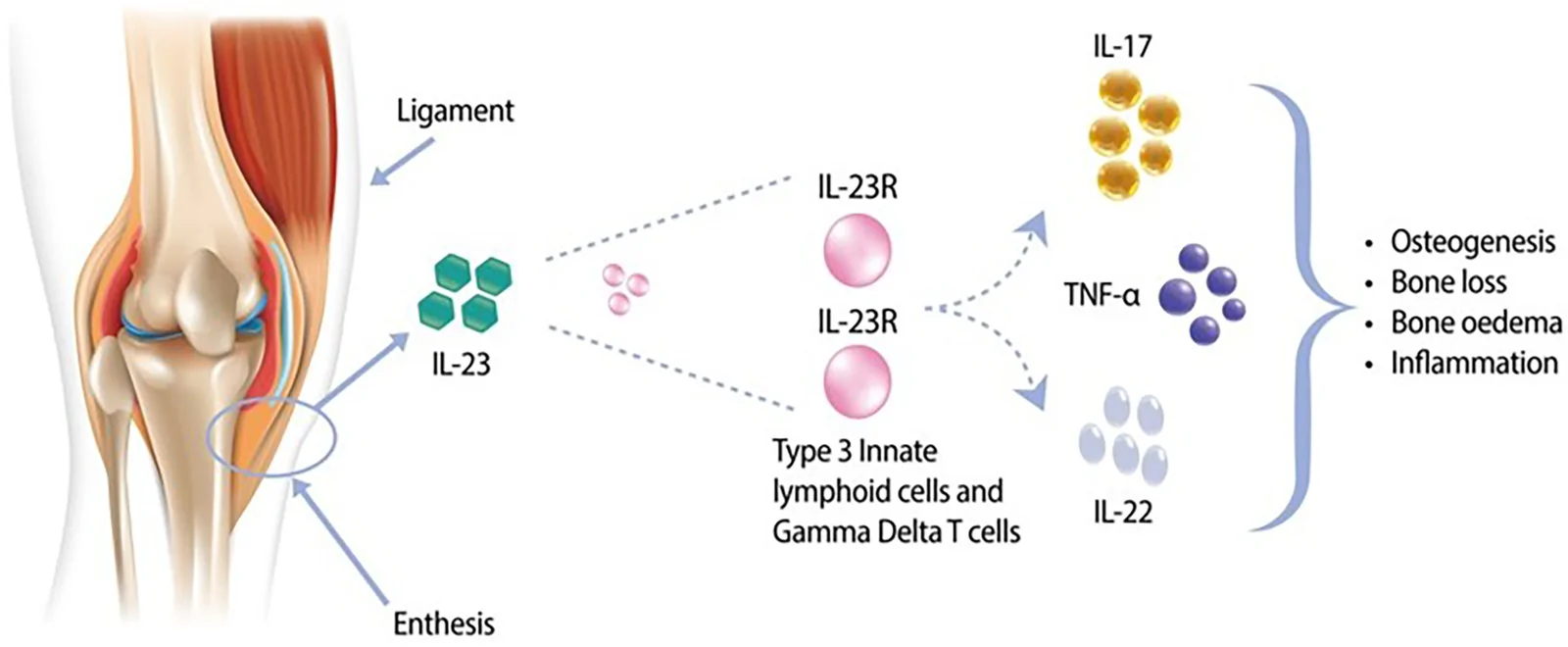
IL-23/IL-23R axis at the enthesis and downstream bone–immune remodeling. Mechanical overload at the ligament–bone insertion (enthesis) induces IL-23, which engages IL-23R on local type-3 immune effectors (e.g., ILC3 and γδ T cells) to drive production of IL-17 and TNF-α (often alongside IL-22). These cytokines amplify inflammation and, together with marrow–enthesis crosstalk via transcortical vessels, promote osteitis and bone-marrow edema while triggering bidirectional bone remodeling—coexisting osteogenesis and focal bone loss. This IL-23 → IL-17/TNF-α axis underlies shared pathology across degenerative enthesopathy and rheumatic enthesitis.

The IL-23/IL-17/TNF-α signaling axis may therefore represent an important mechanistic link between mechanical microdamage and chronic entheseal inflammation, further supporting the concept of a shared disease continuum between degenerative and inflammatory enthesis disorders (Figure 2).

In both conditions, ECM stiffness serves as a pathogenic amplifier, sustaining inflammatory cascades and impairing tissue regeneration. This shared pathophysiological foundation highlights the importance of targeting ECM mechanics—not only for structural restoration but also for immunomodulatory interventions in enthesis-related disorders.

## 3 Subperiosteal lesions and the role of transcortical vessels (TCVs)

The subperiosteal region at the tendon–bone interface plays a pivotal role in enthesopathy. Recent evidence points to transcortical vessels (TCVs), which are microvascular channels traversing cortical bone, as key conduits for immune and inflammatory cell trafficking, linking the bone marrow microenvironment to the enthesis itself.

Bone marrow edema (BME) adjacent to entheses is a common imaging finding not only in inflammatory enthesitis but also in degenerative enthesopathy. MRI studies reveal that BME patterns occur in both SpA-related enthesitis and mechanically induced enthesopathy, suggesting a shared pathophysiological mechanism involving marrow-derived inflammatory processes (22). The underlying mechanism of BME formation involves microdamage to the subchondral and subenthesal bone due to repetitive mechanical loading or inflammation, which increases intraosseous pressure and vascular permeability. This leads to the extravasation of plasma and immune cells into the marrow space, visible as edema-like signals on fluid-sensitive MRI sequences. Additionally, activation of bone-resident immune cells and recruitment of inflammatory cytokines, such as TNF-α and IL-17, further amplifies local inflammation and vascular leakage, reinforcing the cycle of edema and structural remodeling.

**Figure 3:**
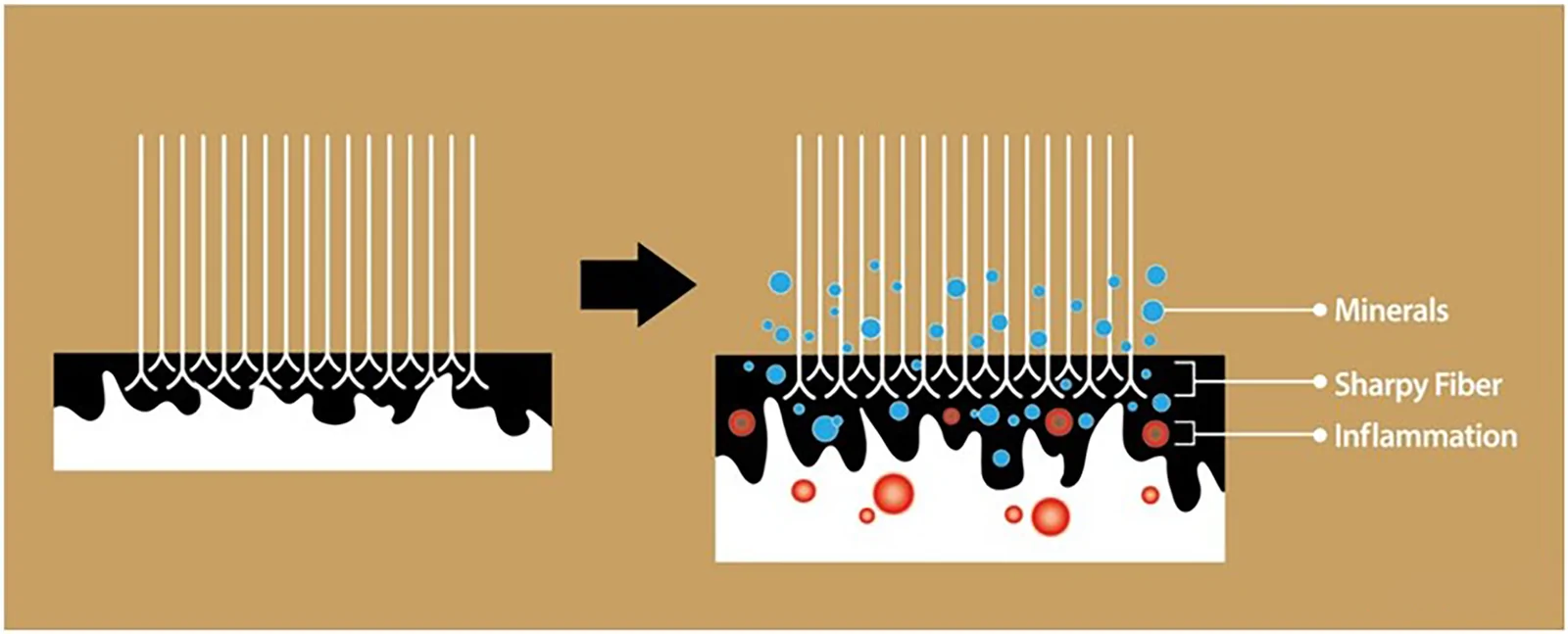
Microdamage-induced mineralization and inflammation at the enthesis. Normal tendon/ligament insertion with vertically oriented Sharpey's fibers anchored into mineralized fibrocartilage and cortical bone (left) and repetitive mechanical load produces micro-tears and fiber disarray (right), leading to focal inflammation (red) within the enthesis and adjacent bone. Concurrently, dystrophic mineral deposits (blue) accumulate along disrupted Sharpey's fibers, promoting enthesophyte formation and stiffening of the insertion. The coupling of inflammation and aberrant mineralization represents an early lesion shared by degenerative enthesopathy and inflammatory enthesitis.

TCVs provide anatomical routes for extravasation of immune cells including neutrophils, macrophages and lymphocytes from the bone marrow into adjacent entheseal and perientheseal tissues. Mechanosensitive translocation via widened TCVs has been postulated to occur following microdamage induced by mechanical overload, effectively serving as a bridge between structural injury and local inflammation (Figure 3).

As mechanical stress at the enthesis increases ECM stiffness and induces microdamage, local vasodilation and TCV engagement can amplify subperiosteal inflammation, leading to osteitis and BME (22). This phenomenon supports the concept that both degenerative and autoimmune enthesopathies share the fundamental pathway of marrow-driven inflammation, mediated by mechanical cues and structural insufficiency.

Ultimately, TCV-mediated immune infiltration and subperiosteal pathology underscore how mechanical stress, ECM rigidity, and immune activation converge at the enthesis, regardless of the upstream trigger. Understanding this shared lesion biology is critical for designing targeted mechanical or pharmacological interventions that address not only ECM restoration but also immune-modulatory pathways.

Collectively, these observations suggest that the enthesis should be viewed not as an isolated insertional structure, but as part of an integrated “bone–ECM–immune axis” in which mechanical stress, vascular communication, and inflammatory signaling continuously interact.

## 4 Commonality in MSC activation and neo-osteogenesis

Mesenchymal stem cells (MSCs), residing in peri-entheseal soft tissues, bone marrow, and the perivascular niche, are highly responsive to mechanical and inflammatory cues. Both degenerative and inflammatory enthesopathies are marked by the aberrant activation of MSCs, which initiate pathological remodeling processes such as fibrocartilage metaplasia and ectopic bone formation. Under sustained mechanical loading or chronic cytokine exposure (e.g., TNF-α, IL-17), MSCs are driven toward chondrogenic and osteogenic lineages, culminating in endochondral ossification and the formation of enthesophytes (in degenerative disease) or syndesmophytes (in inflammatory spondyloarthropathies).

Importantly, ECM stiffness plays an important mechanobiological role in this process. As the local matrix becomes increasingly rigid due to collagen crosslinking, AGE accumulation, or ossification, mechanotransductive signaling via integrins, Piezo channels, and YAP/TAZ is enhanced, thereby promoting chronic inflammation and pathological tissue remodeling. This creates a feed-forward loop that reinforces MSC differentiation into fibrocartilage or bone-forming phenotypes, further exacerbating enthesis ossification. Similar pathological cascades have been observed in both osteoarthritis and ankylosing spondylitis, suggesting shared molecular underpinnings despite differing etiologies.

Recent studies support the notion that mechanical microenvironments govern stem cell fate decisions, with stiff ECMs favoring osteogenesis at the expense of normal enthesis elasticity (23). This convergence of mechanical and immune stimuli on MSC activation represents a key pathological mechanism in enthesis disease, unifying the processes of degenerative and autoimmune ossification under a common biomechanical framework.

## 5 Wolff's Law and regenerative effects of mechanical stimulation

### 5.1 Overview of Wolff's law and Its relevance to ECM dynamics

Wolff's Law posits that bone architecture is not static but dynamically remodels in response to mechanical loading. Traditionally applied to explain trabecular realignment and cortical thickening under stress, this principle has recently been expanded to encompass broader cellular responses in enthesis and peri-entheseal tissues. Low-frequency mechanical loading, particularly in the range of 3–5 Hz, has been shown to stimulate osteoblast differentiation, improve bone mineral density, and initiate favorable ECM remodeling via activation of mechanosensitive receptors such as Piezo1 (24, 25). Importantly, these stimuli do not merely reinforce skeletal structure but also attenuate pathological matrix stiffness by modulating fibroblast activity and inflammatory signaling pathways.

This emerging perspective expands Wolff's Law beyond classical bone adaptation and positions controlled mechanical stimulation as a potential regenerative strategy for ECM-driven musculoskeletal disorders.

### 5.2 Experimental evidence supporting vibration therapy and clinical insights

Emerging preclinical data provide compelling support for the therapeutic utility of vibration-based mechanical stimulation. Li et al. (26) reported that application of low-frequency vibration in rodent models enhanced cortical bone density, promoted Piezo1 expression, and increased osteogenic gene activity (26). Hortobagyi et al. (27) demonstrated that mechanical vibration reduced fibrotic ECM deposition through downregulation of the TGF-β/SMAD signaling axis, a key driver of fibrosis in musculoskeletal tissues (27). Also, recent studies have highlighted the therapeutic potential of periosteal dry needling and low-frequency vibration therapy, both of which apply controlled mechanical stimuli to deep musculoskeletal structures (28, 29). Their findings confirmed significant improvements in ECM compliance, collagen type I/III balance, and clinical symptoms in degenerative enthesopathy models. These results suggest that mechanical vibration, appropriately tuned in frequency and duration, serves as a potent modulator of enthesis homeostasis through both cellular and matrix-level mechanisms.

Targeted mechanical stimulation of periosteal and entheseal regions has been proposed as a mechanobiologically informed intervention grounded in Wolff's Law, emphasizing low-frequency mechanical loading (3–5 Hz) to stimulate subperiosteal and entheseal tissues (24–27).

Drawing upon advances in ECM biology, mechanotransduction research, and clinical rehabilitation practice, this approach has been considered not merely a symptomatic intervention, but a strategy aimed at addressing the biomechanical mechanisms underlying chronic musculoskeletal pain.

This modality has been proposed to modulate ECM stiffness, disrupt pathological collagen cross-linking, and activate mechanosensitive ion channels such as Piezo1, potentially enhancing vascular perfusion, neural recovery, and reducing nociceptive sensitization in fibrotic or hypoxic tissues (24–34).

Application of targeted mechanical stimulation to periosteal and entheseal regions may influence ECM stiffness and tissue compliance across multiple anatomical regions, including the cervical spine, shoulder, lumbar region, pelvis, and knee, particularly when combined with targeted examination, manual therapy, and rehabilitative loading protocols (26, 27, 30–34).

Importantly, this framework suggests that mechanical modulation may help attenuate the stiffness–inflammation cycle associated with chronic musculoskeletal pain (13, 19, 23). By addressing pathological alterations in ECM structure and local mechanical homeostasis, targeted mechanical stimulation of periosteal and entheseal regions may contribute to restoration of matrix compliance and normalization of tissue loading patterns (13, 19, 23). These effects may support functional recovery in patients with chronic musculoskeletal disorders.

This aligns with a growing consensus that regenerative interventions must not only counteract inflammation but also reprogram the mechanical microenvironment, particularly in enthesis-related disorders. Vibration-based therapies thus exemplify a practical and non-invasive application of Wolff's Law principles in clinical musculoskeletal rehabilitation.

## 6 Histological similarities and remodeling patterns

Histopathological studies of enthesis-related disorders have revealed remarkable overlaps between degenerative enthesopathy and rheumatic enthesitis, suggesting a shared underlying mechanism of maladaptive remodeling. In degenerative enthesopathy, histological features include fibrocartilage metaplasia, subperiosteal fibrovascular proliferation, and advancing calcification fronts. These findings are traditionally associated with inflammatory conditions such as ankylosing spondylitis or psoriatic arthritis. These alterations represent an aberrant attempt at tissue repair, where persistent mechanical overload or microdamage promotes a fibrotic and ossifying response instead of regenerative remodeling (35).

Importantly, these changes are not limited to overtly inflammatory diseases. Mechanical stress, acting alone, can initiate a cascade involving ECM stiffening, vascular infiltration, and endochondral ossification within the enthesis and adjacent bone marrow (36). This convergence of mechanical and immune-mediated remodeling underscores a common pathogenic continuum, in which fibrocartilaginous transformation and mineralization represent the endpoint of chronic enthesis dysfunction.

The presence of similar histological hallmarks across both degenerative and autoimmune conditions not only supports the concept of enthesis as a mechanically vulnerable site, but also emphasizes the need for treatment approaches that target ECM stiffness and restore normal remodeling dynamics, regardless of the initiating factor.

## 7 Clinical implications and therapeutic recommendations

Current understanding of enthesis-related disorders increasingly recognizes the interplay between inflammatory signaling and mechanical dysfunction as co-drivers of disease progression. While inflammatory cytokines initiate and amplify pathological cascades within the enthesis, chronic mechanical overload and ECM stiffening exacerbate tissue damage and hinder regeneration. Notably, degenerative enthesopathy often involves both biochemical inflammation and biomechanical disorganization, resulting in limited efficacy of single-modality treatments. Consequently, an integrative therapeutic paradigm that addresses both inflammatory and mechanical contributors has gained traction as a more comprehensive and effective strategy.

### 7.1 Pharmacological intervention: role of corticosteroids in modulating enthesis inflammation

Corticosteroids remain a cornerstone in the pharmacological management of inflammatory musculoskeletal disorders, including enthesopathy. Their potent anti-inflammatory properties can effectively suppress the local production of pro-inflammatory cytokines such as TNF-α, IL-1β, and IL-17, which are frequently elevated in both degenerative and immune-mediated enthesis diseases (37).

When applied judiciously in low doses (≤0.125 mg per site), corticosteroids such as dexamethasone or triamcinolone can offer localized symptom relief while minimizing systemic side effects (38). These micro-dose injections reduce inflammatory cell infiltration, downregulate matrix metalloproteinases (MMPs), and inhibit fibroblast activation thereby interrupting the self-perpetuating cycle of inflammation and matrix remodeling at the tendon–bone interface (39).

However, unlike rheumatic enthesitis, corticosteroids alone often fail to fully resolve degenerative enthesopathy, especially in cases where ECM stiffness is heterogeneous, with localized zones of both fibrosis and residual compliance (40). Fibrotic regions with dense cross-linked collagen may resist steroid penetration and persist as chronic pain generators despite reduced inflammation.

Therefore, steroids are most effective when combined with mechanical interventions that remodel the stiff ECM and restore tissue compliance such as vibration-based therapies or needling techniques. In this integrative approach, corticosteroids suppress the inflammatory drivers, while mechanical stimulation addresses the structural and biomechanical contributors to enthesis pathology.

This combined strategy may offer superior long-term outcomes by promoting matrix normalization, pain relief, and functional restoration, especially in chronic or recalcitrant cases where inflammation alone does not account for the full clinical picture.

### 7.2 Mechanical vibration therapy

Low-frequency mechanical vibration therapy (typically 3–5 Hz) has emerged as a promising non-invasive intervention for modulating ECM stiffness and resolving localized inflammation in enthesis-related disorders. Mechanical stimulation at these frequencies activates deep mechanosensors such as Piezo1 and Piezo2 channels, triggering downstream pathways that promote Sharpey's fiber regeneration, reorganize fibrotic ECM, and attenuate inflammatory signaling.

The therapeutic potential of this approach is supported by empirical findings from two related modalities: periosteal dry needling and low-frequency vibration therapy. Periosteal dry needling involves deep needle insertion near the periosteum and has demonstrated clinical efficacy in conditions such as knee and thumb osteoarthritis. Its mechanisms include local vasodilation, activation of peripheral pain gate control, and suppression of pro-inflammatory cytokines including TNF-α and IL-6 (30, 31).

Similarly, low-frequency vibration therapy, applied at approximately 5 Hz, has shown beneficial effects in osteoarthritis and tendinopathies, possibly through enhanced Piezo-mediated mechanotransduction, increased perfusion, and stimulation of ECM remodeling (32). These modalities indicate that controlled mechanical inputs can effectively reverse matrix stiffening, support enthesis homeostasis, and disrupt the stiffness–inflammation cycle characteristic of degenerative enthesopathy.

Together, these approaches demonstrate that mechanical vibration therapy can serve as a valuable adjunct or alternative to pharmacologic anti-inflammatory interventions, offering both structural and symptomatic relief in ECM-driven musculoskeletal pathology.

Importantly, the therapeutic efficacy of mechanical stimulation likely depends on individualized factors including tissue stiffness, loading history, inflammatory status, and regional biomechanical characteristics, highlighting the need for personalized mechanotherapeutic protocols.

## 8 Discussion

Degenerative enthesopathy represents a multifactorial condition characterized by the convergence of mechanical overload, ECM stiffening, chronic low-grade inflammation, and subperiosteal remodeling. Histological and molecular parallels with rheumatic enthesitis, including fibrocartilage metaplasia, bone marrow edema, and cytokine involvement (e.g., IL-17 and TNF-α), support a unified pathophysiological framework bridging degenerative and autoimmune mechanisms (21, 22, 33, 35). Along similar lines, pathological stiffening of the ECM has emerged as a shared mechanobiological mechanism linking structural degeneration and chronic inflammation (4, 13, 19), which not only disrupts local mechanotransduction but also perpetuates inflammatory signaling.

A key observation from the available literature is that ECM stiffness appears to play a dual role in enthesis pathology. In addition to reflecting cumulative structural damage, increased matrix stiffness may actively contribute to persistent inflammation, altered mechanotransduction, and progressive ossification (13, 19, 23).

Recent advances in mechanobiology and imaging have highlighted the therapeutic potential of Wolff's Law-based mechanical stimulation strategies, such as low-frequency vibration and periosteal mechanical loading, to reverse ECM fibrosis, restore tissue compliance, and re-engage regenerative signaling pathways (24–27, 41).

Interventions involving targeted mechanical stimulation of periosteal and entheseal regions, dry needling, and low-frequency vibration have demonstrated the capacity to modulate mechanosensitive signaling pathways, reduce TGF-β-driven fibrosis, and enhance local vascular and neural regeneration (10, 26–34). These modalities, particularly when combined with adjunctive anti-inflammatory agents or ECM-modulating compounds, offer a minimally invasive approach to treating enthesis-related pain and dysfunction.

Furthermore, emerging regenerative strategies have raised important questions regarding the optimal approach to restoring damaged enthesis tissues. Although mesenchymal stem cell (MSC)-based therapies have attracted considerable attention, increasing evidence suggests that their therapeutic effects are mediated primarily through paracrine mechanisms, particularly extracellular vesicles (EVs) and exosomes, rather than long-term engraftment or direct tissue replacement (39, 42). In contrast, the periosteum and enthesis contain resident skeletal stem/progenitor cell populations that remain viable within their native biomechanical microenvironment and are highly responsive to mechanical cues (43, 44).

Within this context, Bioelectrical Stimulation (BES) can be considered a mechanobiologically relevant intervention that combines localized mechanical and electrical stimulation. BES has been proposed to influence resident progenitor-cell activity, mechanically regulated signaling pathways, and the local biomechanical environment of chronically degenerated entheses (24, 25, 43–46). However, direct evidence supporting BES-induced enthesis regeneration remains limited. Its proposed biological effects should therefore be interpreted cautiously and viewed as hypothesis-generating, pending validation through mechanistic studies and controlled clinical trials.

These observations support the view that degenerative enthesopathy and rheumatic enthesitis share important mechanobiological features despite differences in their initiating mechanisms (13, 21, 35, 47, 48).

Controlled mechanical loading should therefore be considered not only as a potential source of tissue stress but also as a therapeutic input that may contribute to matrix adaptation and repair (24–27, 41). Future studies should focus on optimizing stimulation parameters, integrating stiffness-based diagnostic approaches such as elastography, and defining tissue-specific loading thresholds. Such efforts may help refine future strategies for the management of chronic enthesis-related disorders.

## 9 Conclusion

Degenerative enthesopathy and rheumatic enthesitis have traditionally been regarded as distinct disorders driven by mechanical overload or immune dysregulation, respectively. However, accumulating evidence suggests that both conditions converge through shared mechanobiological pathways involving ECM stiffening, maladaptive mechanotransduction, chronic inflammation, and pathological remodeling.

This review highlights ECM stiffness as an important pathological substrate linking structural degeneration, immune activation, and aberrant ossification at the enthesis. Wolff's Law-based mechanical stimulation strategies may help restore matrix compliance, improve mechanobiological homeostasis, and interrupt the stiffness–inflammation cycle associated with chronic enthesis pathology.

Emerging mechanotherapeutic approaches such as BES may further extend this paradigm by proposing modulation of endogenous periosteal and entheseal progenitor-cell activity through combined mechanical and electrical stimulation. Although clinical validation remains limited, such approaches illustrate how modulation of mechanotransductive pathways may complement anti-inflammatory strategies and contribute to restoration of tendon–bone interface homeostasis (24, 25, 43, 44).

The evidence reviewed here suggests that ECM stiffening is a common feature of both degenerative and inflammatory disorders of the enthesis and contributes to structural deterioration, persistent inflammation, and pathological remodeling. Recognition of these shared biological processes may facilitate the development of therapeutic approaches that address both mechanical and inflammatory aspects of disease.

Further studies are warranted to determine whether interventions targeting matrix stiffness can improve long-term outcomes in patients with chronic enthesis-related disorders.

### 9.1 Future directions

Future research should focus on validating ECM stiffness as a quantitative biomarker for enthesis pathology using advanced imaging modalities such as shear wave elastography and MRI-based tissue characterization.

In addition, further mechanistic studies are required to clarify how mechanosensitive pathways—including Piezo1/2, YAP/TAZ, and TGF-β signaling—interact with inflammatory cascades during chronic enthesis remodeling.

Large-scale clinical trials are also needed to establish standardized protocols for Wolff's Law-based mechanotherapies, including optimal loading frequency, duration, and patient-specific treatment thresholds.

A precision mechanotherapy approach integrating biomechanics, imaging biomarkers, and immunomodulatory strategies may ultimately provide more effective long-term management of chronic enthesis disorders.

Future mechanotherapy studies should also investigate whether bio-electric stimulation approaches can modulate endogenous periosteal progenitor-cell activity and extracellular vesicle signaling through regulation of ECM stiffness and mechanotransductive pathways.

Furthermore, quantitative assessment of tissue stiffness using shear wave elastography, together with biomarkers of mechanotransduction and extracellular vesicle activity, may help establish objective measures for evaluating emerging mechanotherapeutic interventions.
