# Correction: Pathological similarities between degenerative enthesopathy and rheumatic enthesitis: the role of ECM stiffness and Wolff's Law-based mechanical stimulation as a therapeutic strategy

**DOI:** 10.3389/fresc.2026.1999671

**Published:** 2026-09-29

**Authors:** Kang Ahn, Aerisa Ahn, Seung Jun Oh, Hyun Sung Kim

**Affiliations:** 1 Department of Pain and Rehabilitation, Ahnkang Hospital, Seoul, Republic of Korea; 2 Graduate School of Medicine, CHA University, Seongnam-si, Gyeonggi-do, Republic of Korea; 3 Department of Physical Therapy, Sangji University, Wonju-si, Gangwon-do, Republic of Korea

**Keywords:** dry needling, enthesis, enthesopathy, extracellular matrix stiffness, mechanotransduction, vibration therapy

The Generative AI statement was erroneously given as “The author(s) declared that generative AI was not used in the creation of this manuscript.”

The correct Generative AI statement is “The author(s) declared that generative AI was used in the creation of this manuscript. OpenAI ChatGPT, GPT-5 was used only for translation from Korean and language editing. It was not used to generate the scientific content of the manuscript.”

The original version of this article has been updated.

